# Suzuki–Miyaura cross-coupling reaction of 1-aryltriazenes with arylboronic acids catalyzed by a recyclable polymer-supported N-heterocyclic carbene–palladium complex catalyst

**DOI:** 10.3762/bjoc.6.70

**Published:** 2010-06-28

**Authors:** Guangming Nan, Fang Ren, Meiming Luo

**Affiliations:** 1 Key Laboratory of Green Chemistry & Technology of Ministry of Education, College of Chemistry, Sichuan University, Chengdu 610064, P. R. China; 2 Department of Chemistry, Ili Teachers College, Yining 835000, Xinjiang, P. R. China

**Keywords:** 1-aryltriazenes, N-heterocyclic carbene–palladium complex, polymer-supported catalyst, recyclable catalyst, Suzuki–Miyaura reaction

## Abstract

The Suzuki–Miyaura cross-coupling reaction of 1-aryltriazenes with arylboronic acids catalyzed by a recyclable polymer-supported Pd–NHC complex catalyst has been realized for the first time. The polymer-supported catalyst can be re-used several times still retaining high activity for this transformation. Various aryltriazenes were investigated as electrophilic substrates at room temperature to give biaryls in good to excellent yields and showed good chemoselectivity over aryl halides in the reactions.

## Introduction

**Scheme 1:**
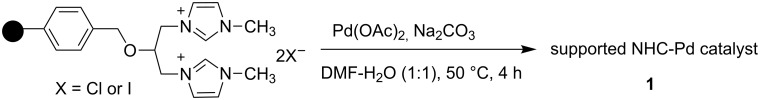
Synthesis of the polymer-supported NHC–Pd catalyst **1**.

The unsymmetrical biaryls feature in a diverse range of organic compounds, such as natural products, advanced materials, liquid crystals, ligands and molecules of medicinal interest [1–4]. The palladium-catalyzed Suzuki–Miyaura cross-coupling reaction has evolved as a powerful synthetic tool for the synthesis of unsymmetrical biaryls in both academic laboratories and industry [5–8]. Most of the reported Suzuki–Miyaura reactions are based on the use of aryl halides and triflates, and recently sulfonates and carboxylates, as the electrophilic component [5–18]. As an additional candidate for the electrophilic coupling partner, arenediazonium salts have also been used in place of aryl halides in the Suzuki–Miyaura cross-coupling reaction, and show higher activity than the corresponding aryl halides [19–28]. However, arenediazonium salts are prone to decompose upon storage, which restricts their practical use. Recently, 1-aryltriazenes, which are stable and can be easily prepared from the corresponding arylamines, have been employed by Tamao et al. as arenediazonium salt surrogates in the Suzuki–Miyaura cross-coupling reaction [29]. Pd_2_(dba)_3_ and P(*t*Bu)_3_ were used as catalyst in this reaction where, as in most of homogeneous catalytic systems, the difficulties of catalyst recovery and recycling constitute major problems. One possible solution to these problems is the ‘heterogenizing’ of a homogeneous catalyst by anchoring the catalyst onto a support. This offers many advantages for industrial application due to their versatile processing capabilities and ease of product/catalyst separation. In view of the potential industrial applications, the development of a recyclable catalytic system for the Suzuki–Miyaura coupling reactions of 1-aryltriazenes is highly desirable. Although several types of heterogenous catalytic systems have been described for the Suzuki–Miyaura reactions of aryl halides [30], to the best of our knowledge, there has been no general study on the Suzuki–Miyaura reactions of 1-aryltriazenes under heterogeneous catalysis described to date. Previously, we reported an active and recyclable polystyrene-supported Pd–NHC (N-heterocyclic carbene) catalyst **1** (Scheme 1) for the Suzuki–Miyaura cross-coupling reactions of aryl bromides, arylsulfonyl chlorides and arenediazonium salts with arylboronic acids that gave biaryls in good to excellent yields [28,31–32]. As part of our ongoing investigations aimed at the development of transition metal catalysis, we report here that the Suzuki–Miyaura cross-couplings of 1-aryltriazenes with arylboronic acids can be readily effected with our polystyrene-supported Pd–NHC catalyst, which shows high efficiency and can be easily recovered and reused several times still retaining high activity.

## Results and Discussion

The polystyrene-supported Pd–NHC catalyst **1** was prepared according to our reported procedure [31] (Scheme 1). The Pd loading was determined to be 0.1 mmol/g by inductively coupled plasma-atomic emission spectrometry (ICP-AES).

**Scheme 2:**
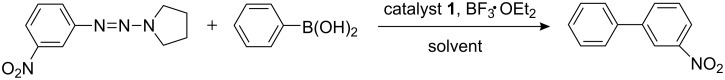
Reaction of 1-(3-nitrophenyl)-2-(pyrrolidin-1-yl)diazene and phenylboronic acid.

The Suzuki–Miyaura cross-coupling of 1-aryltriazenes and boronic acids catalyzed by the polymer-supported Pd–NHC catalyst **1** was investigated in detail with the coupling of 1-(3-nitrophenyl)-2-(pyrrolidin-1-yl)diazene and phenylboronic acid as a model reaction (Scheme 2). As described for the homogeneous catalytic conditions [29], a Lewis acid was essential for the formation of the biphenyl products. Without BF_3_·OEt_2_, no diaryl product was observed whatsoever.

Various solvents were explored for the cross-coupling reaction. As shown in Table 1, solvent significantly affected the product yields. Among the solvents investigated, 1,4-dioxane proved to be the most effective (Table 1, entry 1). DME also led to good yields of the desired product (Table 1, entry 2) whereas THF and CH_3_CN gave lower product yields. No product was formed in either DMF or DMSO.

**Table 1:** Effect of solvent on the cross-coupling reaction^a^.

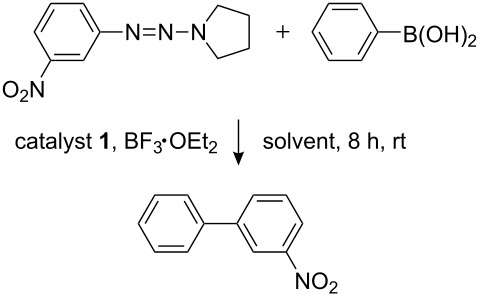		
Entry	Solvent	Yield (%)^b^

1	dioxane	92
2	DME	84
3	THF	42
4	CH_3_CN	46
5	DMF	0
6	DMSO	0

^a^Reaction conditions: 1-(3-nitrophenyl)-2-(pyrrolidin-1-yl)diazene (0.5 mmol), BF_3_·OEt_2_ (0.5 mmol) and phenylboronic acid (1.0 mmol), catalyst **1** (100 mg, 10 μmol Pd), solvent (5 mL), 8 h, rt. ^b^Isolated by silica-gel column chromatography and based on 1-(3-nitrophenyl)-2-(pyrrolidin-1-yl)diazene.

The effect of catalyst loading on the cross-coupling reaction is shown in Table 2. The amount of the polymer-supported Pd–NHC catalyst employed in the reaction is of importance. No product was observed in the absence of catalyst **1** (Table 2, entry 1). The yield of the corresponding biaryl product increased with the catalyst loading (Table 2, entries 2, 3, 4). A high yield of 92% was obtained when 2.0 mol % Pd was employed (Table 2, entry 5) whilst Pd loadings of greater than 2.0 mol % Pd did not lead to improved yields (Table 2, entry 6).

**Table 2:** Effect of the catalyst loading on the cross-coupling reaction^a^.

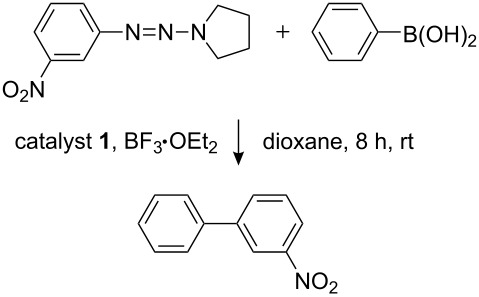		
Entry	Pd (mol%)	Yield (%)^b^

1	0	0
2	0.5	79
3	1.0	83
4	1.5	89
5	2.0	92
6	2.5	92

^a^Reaction conditions: 1-(3-nitrophenyl)-2-(pyrrolidin-1-yl)diazene (0.5 mmol), phenylboronic acid (1 mmol), BF_3_·OEt_2_ (0.5 mmol), catalyst, dioxane (5 mL), rt, 8 h. ^b^Isolated by silica-gel column chromatography and based on 1-(3-nitrophenyl)-2-(pyrrolidin-1-yl)diazene.

Table 3 shows the effect of the molar ratio of phenylboronic acid to 1-(3-nitrophenyl)-2-(pyrrolidin-1-yl)diazene on the reaction yield. A yield of 77% was obtained when the molar ratio was 1.25 (Table 3, entry 1). The yield of the corresponding biaryl product increased with increasing substrate ratio. The highest yield (92%) was obtained when the ratio was increased to 2.0 (Table 3, entry 4) whilst higher substrate ratios did not produce any yield improvement (Table 3, entries 5, 6). Only different reaction rates were observed when the coupling reaction was conducted at different temperatures, which suggested that the reaction temperature did not apparently influence the final reaction yield (Table 3, entry 7).

**Table 3:** Effect of the molar ratio of substrates on the cross-coupling reactions^a^.

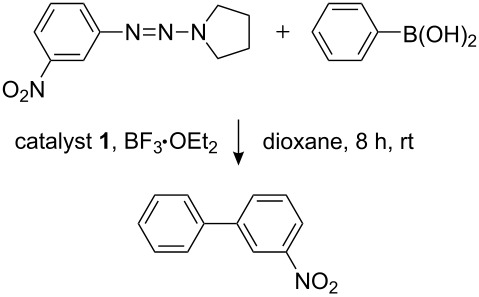		
Entry	Molar ratio^b^	Yield (%)^c^

1	1.25	77
2	1.5	87
3	1.75	90
4	2.0	92
5	2.25	92
6	2.5	91
7	2.0	91^d^

^a^Reaction conditions: 1-(3-nitrophenyl)-2-(pyrrolidin-1-yl)diazene (0.5 mmol), BF_3_·OEt_2_ (0.5 mmol), catalyst **1** (100 mg, 10 μmol Pd), dioxane (5 mL), rt, 8 h. ^b^The ratio of phenylboronic acid to1-(3-nitrophenyl)-2-(pyrrolidin-1-yl)diazene. ^c^Isolated by silica-gel column chromatography and based on 1-(3-nitrophenyl)-2-(pyrrolidin-1-yl)diazene. ^d^At 80 °C for 5 h.

After optimizing the amount of catalyst, solvent, substrate ratio and temperature, the recyclability of the polymer-supported Pd–NHC catalyst for the Suzuki–Miyaura cross-coupling reactions of 1-(3-nitrophenyl)-2-(pyrrolidin-1-yl)diazene and phenylboronic acid was investigated (Table 4). The catalyst could be re-used eight times and still retained high activity after separation, washing and drying under vacuum, under the same reaction conditions. To demonstrate the general recyclability of the catalyst, the recovered catalysts from the first, fifth and eighth runs of the reaction of 1-(3-nitrophenyl)-2-(pyrrolidin-1-yl)diazene and phenylboronic acid were also used to catalyze the reaction of 1-phenyl-2-(pyrrolidin-1-yl)diazene and 4-methoxyphenylboronic acid. The yields were 85%, 80% and 72%, respectively. Contamination with the previous product was not observed in the new product. Analysis of the reaction mixture, following separation and washing of the resin, by ICP-MS indicated that after the initial run (0.4% leaching) there were low levels of Pd leaching (about 90 ppm for the second run and 45 ppm for the fifth run) from the resin. In order to confirm that the reactive catalyst was a solid-supported catalyst rather than a boomerang system, we undertook experiments in which the solid catalyst was filtered off after 2 hours (about 40% conversion), and no further reaction was observed despite stirring for additional 12 hours.

**Table 4:** Recycling of the polymer-supported NHC–Pd catalyst **1**^a^.

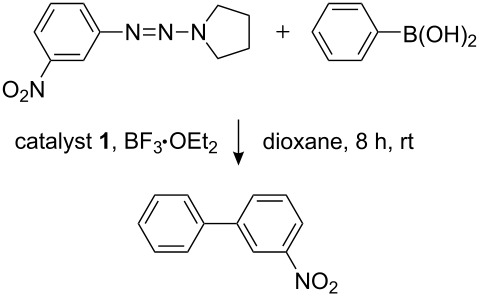								
Run	1	2	3	4	5	6	7	8

Yield (%)^b^	92	92	89	88	85	82	82	78

^a^Reaction conditions: 1-(3-nitrophenyl)-2-(pyrrolidin-1-yl)diazene (0.5 mmol), phenylboronic acid (1.0 mmol), BF_3_·OEt_2_ (0.5 mmol), catalyst **1** (100 mg, 10 μmol Pd), dioxane (5 mL), rt, 8 h. ^b^Isolated by silica-gel column chromatography and based on 1-(3-nitrophenyl)-2-(pyrrolidin-1-yl)diazene.

The scope of the cross-coupling reactions with variety of 1-aryltriazenes and arylboronic acids was then explored under the optimized reaction conditions. As shown in Table 5, most of the cross-coupling reactions afforded biaryl products in good to excellent yields. The electronic nature of substituents and steric factors of both substrates affected the yields of the cross-coupling products. Electron-withdrawing substituents on the 1-aryltriazenes and electron-donating groups on the arylboronic acids gave better yields of biaryl products. Electron-donating groups on the 1-aryltriazenes and electron-withdrawing substituents on the arylboronic acids lead to reduced yields. Steric hindrance of *ortho* substituents slightly reduced the product yields (Table 5, entries 4, 7). Halogens on 1-aryltriazenes gave lower yields (Table 5, entries 5, 6). Notably, good reactivity and chemoselectivity were achieved with 1-(4-bromophenyl)- and 1-(4-iodophenyl)-2-(pyrrolidin-1-yl)diazene (Table 5, entries 5, 6), showing that triazenes were more active than the corresponding aryl bromides and iodides under these reaction conditions, while the homogeneous Pd-phosphine catalyst system gave very low product yields with these substrates [29]. The good reactivity and chemoselectivity may allow haloaryltriazenes to be used as substrates for differential cross-coupling reactions in the same way as haloarenediazonium salts [33].

**Table 5:** Cross-coupling of 1-aryltriazenes and arylboronic acids catalyzed by the polystyrene-supported NHC–Pd catalyst^a^.

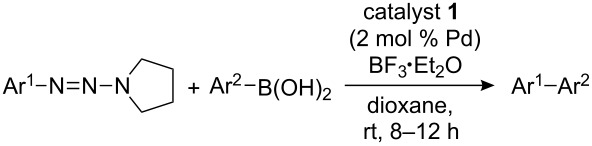				
Entry	Ar^1^	Ar^2^	Time (h)	Yield (%)^b^

1	4-NO_2_C_6_H_4_	C_6_H_5_	8	92
2	2-NO_2_C_6_H_4_	C_6_H_5_	10	87
3	3-NO_2_C_6_H_4_	C_6_H_5_	8	92
4	2-Me-4-NO_2_C_6_H_3_	C_6_H_5_	10	88
5	4-IC_6_H_4_	C_6_H_5_	12	66
6	4-BrC_6_H_4_	C_6_H_5_	12	71
7	1-Naphthyl	C_6_H_5_	12	81
8	4-MeOC_6_H_4_	C_6_H_5_	12	77
9	3-NO_2_C_6_H_4_	4-MeOC_6_H_4_	8	96
10	C_6_H_5_	4-MeOC_6_H_4_	10	84
11	4-MeC_6_H_4_	4-MeOC_6_H_4_	12	82
12	4-NO_2_C_6_H_4_	4-MeOC_6_H_4_	8	96
13	3-NO_2_C_6_H_4_	4-MeC_6_H_4_	8	94
14	4-NO_2_C_6_H_4_	4-MeC_6_H_4_	8	93
15	C_6_H_5_	3-NO_2_C_6_H_4_	12	74

^a^Reaction conditions: 1-aryltriazene (0.5 mmol), arylboronic acid (1.0 mmol), BF_3_·OEt_2_ (0.5 mmol), catalyst **1** (100 mg, 10 μmol Pd), dioxane (5 mL), rt. ^b^Isolated by silica-gel column chromatography and based on 1-aryltriazene.

## Conclusion

In conclusion, we have disclosed that the Suzuki–Miyaura cross-coupling of aryltriazenes and arylboronic acids can be catalyzed by the recyclable polystyrene-supported Pd–NHC catalyst **1** to produce biaryls in good to excellent yields. The supported Pd–NHC catalyst can be re-used several times and still retains high activity, which reflects its high stability and recyclability. Advantages of this method also include good substrate generality, good chemoselectivity over arylhalides and mild reaction conditions.

## Experimental

### General

All chemicals were obtained from commercial sources and were, in general, used without further purification. Melting points were determined with XRC-1 melting point apparatus and were uncorrected. ^1^H NMR spectra were recorded on Varian INOVA 400 MHz or Bruker Avance 600 MHz spectrometer. GC-MS were recorded on an Agilent 5973 N. Palladium content was determined by ICP-AES on IRIS Adv. Pd-leaching was determined by ICP-MS on VG PQ Exceu.

### General procedure for the preparation of 1-aryltriazenes

1-Aryltriazenes were prepared by a modification of the literature procedure [34]. A solution of arylamine (10 mmol) in concentrated HCl (2 mL) was cooled in an ice bath while a solution of NaNO_2_ (10 mmol) in cold water (1 mL) was added dropwise. The resulting solution of the diazonium salt was stirred in the cold for 10 min and then added all at once to a chilled solution of pyrrolidine (11 mmol) in 1 M KOH (10 mL). The reaction mixture was stirred for 30 min with cooling and the resulting precipitate isolated by filtration. The damp solid was recrystallized from EtOH and dried under reduced pressure.

### General procedure for the Suzuki–Miyaura cross-couplings of 1-aryltriazenes and arylboronic acids

Polystyrene-supported Pd–NHC catalyst **1** (100 mg, 10 μmol Pd), 1-aryltriazene (0.5 mmol), arylboronic acid (1 mmol) were mixed in dioxane (5 mL). The mixture was stirred and BF_3_·OEt_2_ (65 μL, 0.50 mmol) added dropwise at room temperature under an argon atmosphere. When the reaction was complete, the catalyst was filtered, washed with ether (5 mL × 3), and then dried under vacuum for the next run. After evaporation of the solvent from the filtrate under reduced pressure, the product was purified by silica gel column chromatography.

**3-Nitrobiphenyl** [35]: yellow solid, mp 59–60 °C. ^1^H NMR (400 MHz, CDCl_3_): δ 7.41–7.51 (m, 3H), 7.59–7.63 (m, 3H), 7.91 (dt, *J* = 8.0 Hz, 1.2 Hz, 1H), 8.18–8.20 (m, 1H), 8.45 (s, 1H). ^13^C NMR (100 MHz, CDCl_3_): δ 148.7, 142.9, 138.7, 133.1, 129.7, 129.2, 128.8, 128.6, 127.2, 122.1, 122.0. MS (ESI): *m/z* 119.1 (M^+^).

**4-Iodobiphenyl** [36]: white solid, mp 112–113 °C. ^1^H NMR (400 MHz, CDCl_3_): δ 7.27–7.38 (m, 3H), 7.44 (t, *J* = 7.4 Hz, 2H), 7.55 (d, *J* = 7.6 Hz, 2H), 7.76 (d, *J* = 8.4 Hz, 2H). ^13^C NMR (100 MHz, CDCl_3_): δ 140.7, 140.1, 137.9, 129.0, 128.9, 127.7, 126.9, 93.0. MS (ESI): *m/z* 280.0 (M^+^).

The structures of the other cross-coupling products, 2-nitrobiphenyl [37], 4-nitrobiphenyl [37], 4-methoxybiphenyl [38], 1-phenylnaphthalene [39], 2-methyl-4-nitrobiphenyl [40], 3-nitro-4′-methylbiphenyl [41], 4′-methoxy-4-methylbiphenyl [12], 4′-methoxy-3-nitrobiphenyl [37], 4-bromobiphenyl [36], 4′-methoxy-4-nitrobiphenyl [38], 4′-methyl-4-nitrobiphenyl [42] were confirmed by comparing their ^1^H NMR ^13^C NMR spectra and melting points with the data and values reported in literature.

### Inductively coupled plasma-atomic emission spectrometry (ICP-AES)

The polystyrene-supported NHC–Pd catalyst **1** (10 mg) in a porcelain crucible was heated at 600 °C in a muffle furnace until there was constant weight. The residue in the crucible was treated with a mixture (5 mL) of hydrochloric acid and nitric acid (3:1, v:v) at 100 °C for 4 h. The resulting solution was diluted to 50 mL with distilled water and analyzed by ICP-AES. The Pd loading was determined to be 0.1 mmol/g.

### Inductively coupled plasma mass spectrometry (ICP-MS)

When the reaction was completed, the catalyst was filtered and washed with ether (3 × 5 mL). The combined organic phase was evaporated under reduced pressure. The residue was heated in a crucible at 600 °C and ignition continued until constant weight and the residue treated with a mixture (5 mL) of hydrochloric acid and nitric acid (3:1, v:v) at 100 °C for 4 h. The resulting solution was diluted to 50 mL with distilled water and analyzed by ICP-MS.

## References

[1] Hassan J, Sévignon M, Gozzi C, Schulz E, Lemaire M (2002). Chem Rev.

[2] Kohta S, Lahiri K, Kashinath D (2002). Tetrahedron.

[3] Littke A F, Fu G C (2002). Angew Chem, Int Ed.

[4] Stanforth S P (1998). Tetrahedron.

[5] Miyaura N, Miyaura N (2002). Organoboron compounds. Cross-coupling reactions.

[6] Miyaura N (2002). J Organomet Chem.

[7] Suzuki A (1999). J Organomet Chem.

[8] Miyaura N, Suzuki A (1995). Chem Rev.

[9] Kwong F Y, Chan K S, Yeung C H, Chan A S C (2004). Chem Commun.

[10] Zhang C, Trudell M L (2000). Tetrahedron Lett.

[11] Kwong F Y, Lam W H, Yeung C H, Chan K S, Chan A S C (2004). Chem Commun.

[12] Navarro O, Kelly R A, Nolan S P (2003). J Am Chem Soc.

[13] Nguyen N H, Huang X, Buchwald S L (2003). J Am Chem Soc.

[14] Gooβen L J, Gooβen K, Stanciu C (2009). Angew Chem, Int Ed.

[15] Guan B-T, Wang Y, Li B-J, Yu D-G, Shi Z-J (2008). J Am Chem Soc.

[16] Quasdorf K W, Tian X, Garg N K (2008). J Am Chem Soc.

[17] Quasdorf K W, Riener M, Petrova K V, Garg N K (2009). J Am Chem Soc.

[18] Antoft-Finch A, Blackburn T, Snieckus V (2009). J Am Chem Soc.

[19] Darses S, Jeffery T, Genêt J P, Brayer J L, Demoute J P (1996). Tetrahedron Lett.

[20] Sengupta S, Bhattacharyya S (1997). J Org Chem.

[21] Darses S, Genêt J P, Brayer J L, Demoute J P (1997). Tetrahedron Lett.

[22] Darses S, Michaud G, Genêt J P (1999). Eur J Org Chem.

[23] Andrus M B, Song C (2001). Org Lett.

[24] Selvakumar K, Zapf A, Spannenberg A, Beller M (2002). Chem–Eur J.

[25] Dai M, Liang B, Wang C, Chen J, Yang Z (2004). Org Lett.

[26] Gallo V, Mastrorilli P, Nobile C F, Paolillo R, Taccardi N (2005). Eur J Inorg Chem.

[27] Roglans A, Pla-Quintana A, Moreno-Mañas M (2006). Chem Rev.

[28] Qin Y, Wei W, Luo M (2007). Synlett.

[29] Saeki T, Son E C, Tamao K (2004). Org Lett.

[30] Zeng X, Zhang T, Qin Y, Wei Z, Luo M (2009). Dalton Trans.

[31] Kang T, Feng Q, Luo M (2005). Synlett.

[32] Zhang S, Zeng X, Wei Z, Zhao D, Kang T, Zhang W, Yan M, Luo M (2006). Synlett.

[33] Sengupta S, Sadhukhan S K (1998). Tetrahedron Lett.

[34] Margaret L G, David H B, Willard M W (1993). J Org Chem.

[35] Pourbaix C, Carreaux F, Carboni B (2001). Org Lett.

[36] Cella R, Cunha R L O R, Reis A E S, Pimenta D C, Klitzke C F, Stefani H A (2006). J Org Chem.

[37] Li J H, Liu W J (2004). Org Lett.

[38] Denmark S E, Ober M H (2003). Org Lett.

[39] Wolf C, Ekoue-Kovi K (2006). Eur J Org Chem.

[40] Iihama T, Fu J-M, Bourguignon M, Snieckus V (1989). Synthesis.

[41] Hassan J, Hathroubi C, Gozzi C, Lemaire M (2001). Tetrahedron.

[42] Kitamura Y, Sakurai A, Udzu T, Maegawa T, Monguchi Y, Sajiki H (2007). Tetrahedron.

